# Replantation of multiple digits and hand amputations: four case reports

**DOI:** 10.1186/1757-1626-1-266

**Published:** 2008-10-23

**Authors:** Mohammed Murshid Salah, Khalid N Khalid

**Affiliations:** 1 Plastic and Hand Surgery Unit, Hamad Medical Corp, Doha, PO box 3050, Qatar

## Abstract

This study reports four cases of hand avulsion at the proximal wrist level and multiple digits amputation were received in plastic and hand surgery unit during the year 2007–2008. All patients were male labors between 22–30 years old, and the amputation due to machine injuries. Successful replantation were achieved, after a period of follow up with occupational therapy all patients regain good functional and cosmetic results. This study proves the strong indication of replantation of multiple digits & hand amputations.

## Background

Digital & hand replantation first became a reality in the 1960s with the advent of microsurgical techniques. Indications for replantation have evolved over the ensuing years and currently include hand amputation, thumb amputations, multiple digit amputations, and amputations in children. Crush and avulsion injuries and amputations of a single digit proximal to the flexor digitorum superficialis insertion remain relative contraindications. Good communication between the replantation center micro surgeon and the referring physician is paramount to achieving appropriate and timely referrals and correct transport of amputated parts. Communication with patients is also important: possible candidates for replantation must be informed of the likely outcomes of replantation and revision amputation procedures, and the different postoperative regimens for each. For patients who choose revision amputation or whose replants do not survive, there are a variety of reconstructive options available, if necessary, such as toe-to-hand transfer. The techniques to perform such elective free tissue transfers have been perfected during the last 30 years largely from experience gained through digital replantation. Even though replantation surgery has now become a routine procedure, it remains delicate and demanding surgery, requiring adequate training and expertise in microsurgical techniques. Although replantation procedures have been simplified, a second surgical team can save valuable surgical time by debriding and identifying the vessels in the amputated part, harvesting microvenous grafts, and performing bone fixation or tendon repair among other things, while the chief surgeon focuses on revascularization. Overall, the most significant guideline underlining the philosophy of digital & hand replantation today reflects the aim of not only ensuring the survival of a digit, but its functional use as well. Experience dictates that this can be achieved only if the basic principles and indications of replantation surgery are adhered to. Replantation of multiple digits & hand remains a daunting challenge that presents especially if it is avulsion-crush injury. The hand Crushing, pulling and rotating forces are responsible for the most severe injuries to an amputated parts and are often contraindications for replantation. Under these circumstances, the extent of tissue damage is difficult to assess, particularly in vessels and nerves. Nerve fibers may be damaged at various levels and stretched vessel walls manifest as a 'ribbon sign'. Both may be found in several locations and at various distances from the actual severing point. The aim of the study is to present the author's experience in primary treatment of patients with fingers and hand amputation. Suggestions for management are given. A high survival rate of replanted tissues and good functional results were achieved despite of the severity of trauma [1].

## Patients and methods

The amputated extremity should be viewed as a distraction to initial evaluation. The patient's vital signs, general health assessment, and general physical examination should be assessed and addressed first. Then the amputation stump is wrapped in a gauze dressing. Bleeding, when present, is addressed with compression. Tourniquets are not used! Since wrist-proximal amputations involve muscle, ischemic time is particularly important. Whereas digits may be routinely replanted with up to 24 hours of cold ischemia time and 12 hours of warm ischemia time, wrist proximal amputations should be performed before 12 hours of cold ischemia time or 6 hours of warm ischemia time have elapsed [2]. Therefore, as part of the initial treatment, ambulance personnel and emergency physicians should be counseled to wrap the amputated part in gauze in plastic package, and cooled on ice. Not inside the ice bag because the hypotonic water may cause water intoxication & cell damage. Once this is done, decisions regarding the feasibility of revascularization or replantation are considered. The description of the mechanism of the injury is critical [3]. Those patients with broad crush or avulsion injuries are typically poor candidates for replantation, whereas amputations resulting from sharp objects are the ideal candidates for replantation because the zone of injury is largely confined to the amputated site. Sufficient bone must be available for stable fixation, and the lacerated vessels, although frequently requiring interposition grafts, must have retained their distal capillary integrity [4]. In the absence of these conditions, successful replantation is impossible and the patient should be informed of this reasoning. In addition, the referring hospital and the patient should be informed that those patients with incomplete amputations far better than complete amputations; Blomgren and colleagues identified decreased operative time, reduced postoperative morbidity, and a 92% successful reconstruction in the former versus prolonged operative time, increased morbidity, and a 71% success rate in the later. A pertinent medical history must be obtained during this process. Cardiac, pulmonary, and neurologic status must be weighed against the stress of transport and subsequent surgery. For example, a recent myocardial infarction or dementia would serve as absolute contraindications for replantation surgery. Strict guidelines for other conditions such as diabetes, renal failure, or a prolonged history of nicotine consumption do not exist. The patient needs to be informed that the complication rate is higher and the success rate lower with these conditions and a lengthy operation and hospitalization may end in failure. Social history is also relevant to the decision. The patient's age, occupation, and social situation influence the aggregate candidacy of the patient for replantation. A return to gainful employment is usually greater than 6–12 months in manual laborers, a period of time that may be unacceptable to a self-employed tool user such as a carpenter, farmer, or rancher. Given no contraindications, expeditious transport must then be arranged; air transport is the standard method for long-distance referrals and ground transport for more local referrals. In those patients where the transport time is prolonged, a temporary vascular shunt may be helpful. Shunts are particularly useful for patients with incomplete amputations. An intact skin bridge or a single intact vein is ideal, while arterial flow is reconstituted with a carotid shunt or a large intravascular catheter [5]. This procedure should be performed in the controlled setting of an operating room and once placed the shunts should be secured with silk sutures or "vessel helpers". In cases of complete amputations a venous and arterial shunt are essential or the patient may exsanguinate during the transportation. The risk of exsanguination even with arterial and venous shunts is high enough that we advise using this technique with caution. Patients with shunt procedures will require blood transfusion and should be transported with 4 units of typed and cross-matched packed red blood cells (PRBCs). Most importantly, the efforts described above need to be weighed against the overlying theme that no single extremity is worth a patient's life and these shunting efforts as well as replantation may prove impractical. In addition, the time delay for the shunt procedure may override the benefits of revascularization.

Replantation may be contraindicated for reasons involving the patient or the digit.

Patients may decline on the basis that they want to return to work rapidly. Cosmetic concerns will determine many decisions. Age negatively affects the digit's capacity to recover, especially sensation, and it affects the patient's ability and will to rehabilitate. Sophisticated hand function and cosmetic considerations are less relevant in the elderly, and many will elect not to replant. On the other hand, elderly patients' hands are relatively stiff and compromised so that loss of digits will theoretically affect them more. Replanted digits in such hands, even if suboptimal, may closely approximate preinjury status and be highly prized. Medical fitness for prolonged anesthesia and prolonged rehabilitation also must be considered. Major associated injuries may mitigate against replantation as would uncooperative patients, for example, with mental retardation who may not tolerate postoperative care and rehabilitation. Relatives, especially when a child is involved, ask if they could donate their finger, but rejection of course contraindicates this. Rejection was on the mind of surgeons who were confronted with a bizarre case in Argentina [Loda G, personal communication, 2004]. In a tug of war, the rope broke leading to avulsion amputation of 20 or more digits from multiple participants. A well intentioned but unthinking bystander collected the digits, placed them on ice in a single plastic bag and proudly brought them to the emergency department where the spectacle was reported of amputees frantically squabbling over identification and ownership of their parts.

Grossly damaged digits or inappropriate proximal stumps are the usual contraindications for replantation. Multiple-level injuries and severe avulsions involving tendons and nerves will not function, and digits that have prolonged ischemia times or are frozen are unlikely to revascularize. Even though a digit may be judged non replantable in the case of multiple amputation, a unique opportunity for improvisation exists where the least damaged amputated digits may be replanted on the most useful stump to provide a digit with better function than if replantation proceeded in its true position. It may be better to obtain one finger functioning well in an optimal state than to have two less functional digits. A decision to replant often is not made until all structures have been identified and the extent of the damage recognized clearly. Besides digital transposition, digits unsuitable for replantation can provide vascularized small joints for transfer, nerves for grafting, and innervated or venous free flaps. In bilateral injuries, cross hand digital transfers can be considered to maximize function or be used to improve function in previously injured dominant digits.

After surgery, the patient is kept in a warm room to promote vasodilation for 3 to 5 days. Hematocrit, parameters for disseminated intravascular coagulopathy (DIC), and electrolyte balance are carefully monitored. An hematocrit of 20 to 25 is ideal and electrolyte balance is kept close to normal. Intravenous fluids are delivered at a rate of twice maintenance rate for 3 days, then routine maintenance rates thereafter. As pointed out by Askari and colleagues, there are inadequate data to develop a rational evidence-based approach to anticoagulation in the setting of microsurgery [6]. We start low molecular weight dextran (Dextran-40) intraoperatively and if there are no untoward reactions, the dextran is continued for the next 3 to 5 days. One baby aspirin (85 mg) is given daily while subcutaneous heparin, 5000 units twice daily, are given to address potential lower extremity deep venous thrombosis associated with bed rest. Therapeutic anticoagulation with heparin and warfarin is reserved for the most desperate situations and is associated with a significantly larger blood loss and risk to the patient. Hourly monitoring with pulse-oximetry and capillary refill is conducted until the patient is discharged. In 48 to 72 hours the patient is returned to the operating room for a dressing change and further debridement if necessary and definitive soft tissue closure, which may require skin grafting or the use of flaps. The patient is returned to the ward and monitored for 3 to 4 days if no grafts or flaps are necessary and 6 to 10 days if flaps are required.

### Patient 1

This is a 30 years old right-handed male labor, while cleaning a cement mixing machine his left hand was trapped in the machine sustained crush avulsion amputation at the intercarpal joint level of the wrist 1. The tendons avulsed at the tenomuscular junction. After resuscitation in accident & emergency unit, patient was transferred to hand unit. Replantation performed 6 hours after the accident. Two teams worked to prepare the vessels & nerves & other structures. Intercarpal arthrodesis, ulnar artery anastomosis basilic vein reconstruction with vein graft. Flexor & extensor tendons were reinserted to appropriate muscles, wounds were covered with split thickness skin graft. Post-operative period was uneventful. Median nerve was reconstructed by cable graft from sural nerve later on. The Patient developed protective sensation on palm and in all fingers 1 year after the accident. He uses the replanted hand in basic every day activities and some more precise activities. Hand grip strength is 25 kg.

**Figure 1:**
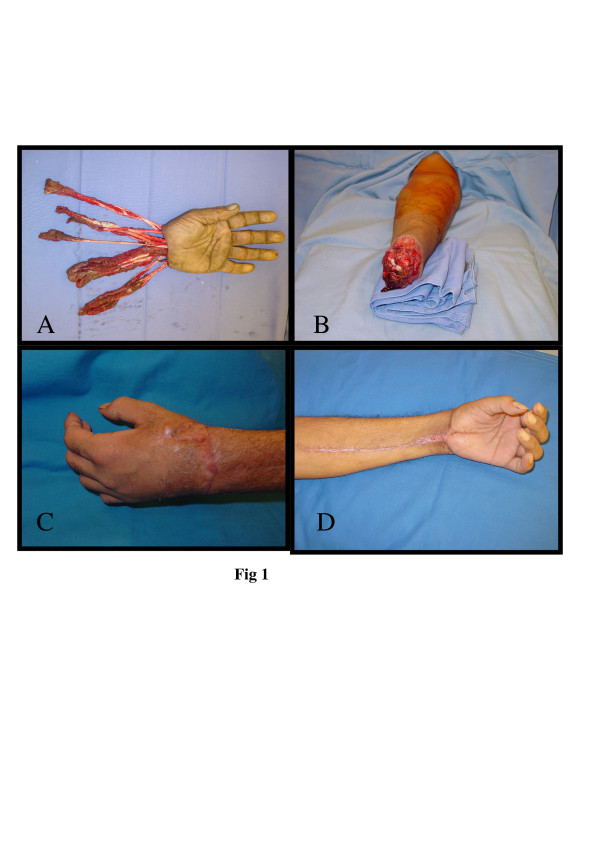
A&B: Avulsion amputation at wrist level with tendons avulsed at tenomuscular junction. C&D: postoperative results.

### Patient 2

This is 27 years old right handed male steel cutter, his left hand was cut by electric saw sustained total amputation at the metacarpophalangeal joint of the four medial fingers & thumb at the interphalangeal joint 2. After period of resuscitation in accident & emergency he arrived to the hand unit after 4 hours of the accident with the amputated finger wrapped with wet gauze in ice bag. the metacarpal heads were fixed by k wires, micro vascular anastomosis to digital arteries & veins were done. Tendons were repaired by modified kessler technique.

**Figure 2:**
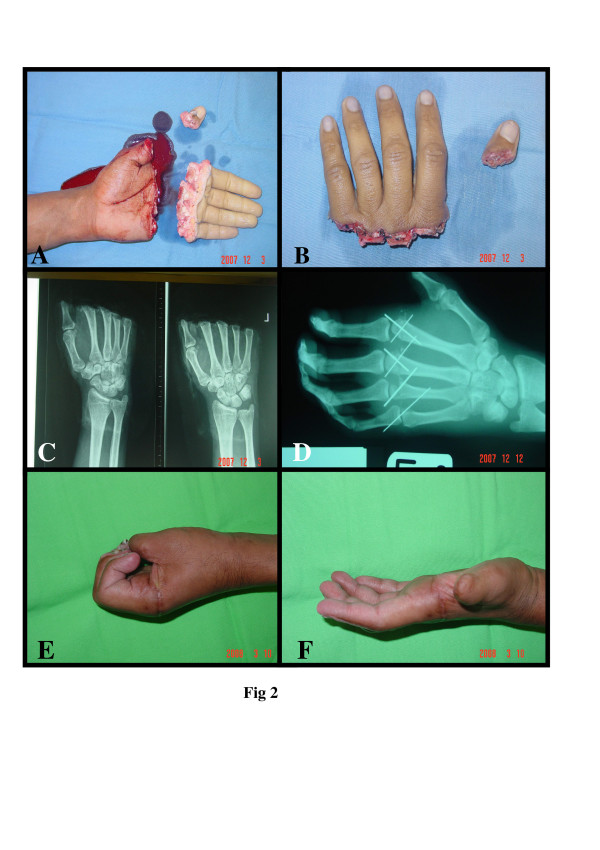
Selection of teams.

### Patient 3

This is 22 years old right handed male carpenter, his left hand was cut by electric saw sustained total amputation of his left ring finger just proximal to proximal interphalangeal joint & near total amputation of middle finger with cut both neurovascular junction at level of mid part of middle phalanx & open comminuted fracture of middle phalanx little finger with cut ulnar neurovascular bundle & tip injury thumb 3. Replantation of the ring, revascularization of middle & open reduction internal fixation of the fractures was done After a period of rehabilitation for three months patient was back to his original work.

**Figure 3:**
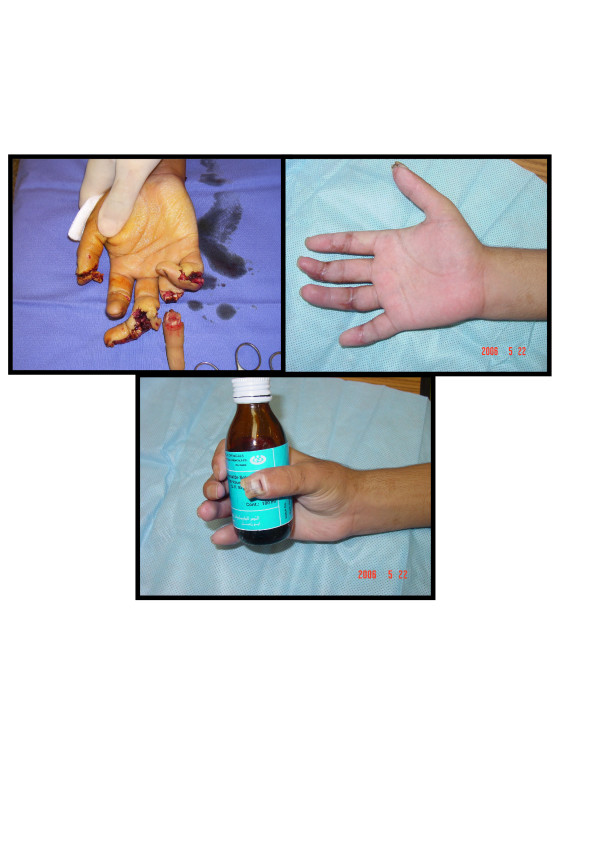
Upper left: Multiple finger amputations & near amputations at different levels. Upper right & lower: post-replantation & revascularization functional & cosmetic results.

### Patient 4

This is a 24 years old right-handed male carpenter, his left hand was cut by electric saw sustained amputation of the thumb at the metacarpophalangeal joint and the index middle finger at the same level and the ring finger at the mid part of the proximal phalanx successful replantation was done and the operation took about 8 hours and was done under loupe magnification 4. After a period of occupational therapy the patient regain good cosmetic and functional results.

**Figure 4:**
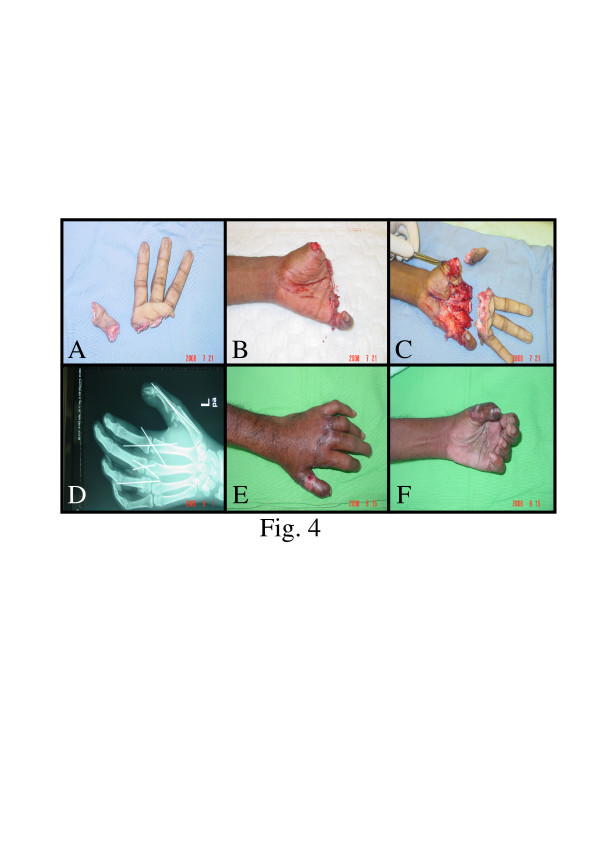
**A&B&C: Multiple fingers clean cut amputations at the metacarpophalangeal joints level. D: X-ray pre-operative. E&F: Final results after seven weeks**.

## Discussion

With the evolution of surgical techniques and scientific technology, the field of replantation has become more refined and specific indications for replantation, protocols for preparation, efficient techniques to ultimately minimize ischemia times and improve survival rates, guidelines for postoperative care, strategies for treating complications, and goals for outcomes have been established. Patient satisfaction hinges on their level of expectation as defined and explained in the preoperative discussion and informed consent process. When considering multiple-finger replantation, the finger with the best chance for successful replantation, best expected recovery, and most significant contribution to function should be repaired first. If all the fingers are injured equally and have the same chance for successful repair, the authors prefer to repair the middle, then index, then ring, and, lastly, the small finger. If the index finger is stiff or insensate, the patient bypasses this to use the middle finger. When all the fingers are stiff, the index finger can actually impede the function and opposition of the other fingers to the thumb. Each finger is replanted separately to minimize warm ischemia time. Minimizing ischemia time is essential when performing multiple-digit replantations. The amputated fingers should be brought to the operating room as soon as possible because the digital vessels, nerves, and tendons can be identified and tagged with sutures or clips, which saves time and minimizes ischemia. The order for repairs can be improvised with multiple replantations. Initially, the osteosynthesis, extensor tendon, one dorsal vein, and one digital artery can be repaired for each finger to minimize overall ischemia time, then another dorsal vein, the digital nerves, and the flexor tendon core sutures can be repaired later, once blood flow has been reestablished to the fingers. Successful replantation is no longer measured by survival of the amputated or devascularized part, but rather by function of that part. Although numerous reports attest to successful replantation and revascularization in the upper extremity, there has been little discussion of functional outcome, especially at the transmetacarpal level. In reviewing of one series of transmetacarpal injuries, They found discouragingly poor long-term functional results and frequent need for secondary surgery. These poor results were not the result of failure of replantation or revascularization technique, but rather due to tendons adhesions, joint contractures, intrinsic tightness, and poor return of sensibility. In our cases, range of motion, pinch and grip strengths, intrinsic muscle function, return of sensibility, and functional recovery according to Chen et al.'s criteria were good. Meyer et al. noted the problem of intrinsic tightness in their original case report. Subsequent reports by Russell et al. 5 and Tark et al. have documented weak or absent intrinsic muscle function after trans-metacarpal injuries. Scheker et al. [Scheker et al., 1994] cited favorable results after four transmetacarpal replantations, which they attributed to (1) resection of the devascularized and denervated intrinsic muscles distally to allow the intrinsic tendons to tenodese in an intrinsic-plus position and (2) a postoperative protocol initiated 72 hours after replantation consisting of early protective active mobilization with anticlaw splinting. Although their preliminary results are encouraging, their account is limited to only four patients, three with guillotine-type injuries. Based on our experience and review of the literature, we see the main factors contributing to the poor functional results after transmetacarpal injuries as being (1) intrinsic muscle ischemia due to either direct muscle injury or interruption of their delicate blood supply and (2) creation of a "common wound." Both of these are unique to the particular location of the injury. In crush injuries with an extensive zone of injury, the intrinsic muscles suffer irreparable damage; the resultant fibrosis and scaring result in a high incidence of intrinsic-related complications. Even in guillotine-type injuries, intrinsic tightness plays a major role in the poor functional results as noted. Therefore, the extent and mechanism of injury were not the only critical factors in the functional outcome. Could the critical injury therefore be ischemia? Disruption of the normal vascular anatomy of the hand plays an important dual role. It is responsible for both the high incidence of digital survival in transmetacarpal replantation and the poor function associated with disruption of the blood supply to the intrinsic muscles that cannot be reconstituted even with successful distal revascularization. Nakamura et al. s reported a successful four-finger transmetacarpal. In our cases early post operative occupational therapy plays major role in our good functional results. Patients started passive movement 2^nd ^post operative day so the chance of adhesions is less.

## Conclusion

Despite the fact that functional outcome of replanted hands & fingers will never equal that of the normal healthy counterpart, replantation has major functional, cosmetic and psychological benefits. Our patients were very satisfied with their replanted hands, which have helped them to return to a better quality of life than they might otherwise have had. We found hand & multiple finger amputations are strong indication for replantation even if it is not indicated for single digit like crush avulsion injury.

## Competing interests

The authors declare that they have no competing interests.

## Authors' contributions

Case 1 was done by KNK assisted by MMS while remaining cases were done by MMS. MMS was involved in preparing the manuscript and both authors reviewed the article.

## Consent

Written consent was obtained from each of the patients.
